# Correlation between supine flexibility and postoperative correction in adolescent idiopathic scoliosis

**DOI:** 10.1186/s12891-023-06227-x

**Published:** 2023-02-15

**Authors:** Mingzheng Zhang, Wenxuan Chen, Shengru Wang, You Du, Jianguo Zhang, Fang Pu

**Affiliations:** 1 Advanced Innovation Center for Biomedical Engineering, School of Biological Science and Medical Engineering, Beihang University, Beijing, China; 2 Beijing Key Laboratory of Rehabilitation Technical Aids for Old-Age Disability, Key Laboratory of Neuro-Functional Information and Rehabilitation Engineering of the Ministry of Civil Affairs, National Research Center for Rehabilitation Technical Aids, Beijing, China; 3 The Department of Orthopedic Surgery, Peking Union Medical College Hospital, Peking Union Medical College and Chinese Academy of Medical Sciences, Beijing, China

**Keywords:** Adolescent idiopathic scoliosis, Supine flexibility, Postoperative correction, Supine radiographs, Scoliosis assessment

## Abstract

**Background:**

The preoperative flexibility of the scoliotic spine is a key aspect of surgical planning, as it provides information on the rigidity of the curve, the extent of structural changes, the levels to be fused and the amount of correction. The purpose of this study was to assess whether supine flexibility can be used to predict postoperative correction in patients with adolescent idiopathic scoliosis (AIS) by determining the correlation between these two characteristics.

**Methods:**

A total of 41 AIS patients who underwent surgical treatment between 2018 and 2020 were retrospectively enrolled for analysis. Preoperative and postoperative standing radiographs and preoperative CT images of the entire spine were collected and used to measure supine flexibility and the postoperative correction rate. T tests were used to analyse the differences in supine flexibility and postoperative correction rate between groups. Pearson’s product-moment correlation analysis was performed, and regression models were established to determine the correlation between supine flexibility and postoperative correction. Thoracic curves and lumbar curves were analysed independently.

**Results:**

Supine flexibility was found to be significantly lower than the correction rate but showed a strong correlation with the postoperative correction rate, with *r* values of 0.68 for the thoracic curve group and 0.76 for the lumbar curve group. The relationship between supine flexibility and postoperative correction rate could be expressed by linear regression models.

**Conclusion:**

Supine flexibility can be used to predict postoperative correction in AIS patients. In clinical practice, supine radiographs may be used in place of existing flexibility test techniques.

## Introduction

Assessment of the preoperative flexibility of the scoliotic spine is important for surgical planning, as it provides information on the rigidity of the curve, the selection of fusion levels and the amount of correction [1–6]. Flexibility is determined by dividing the difference in the Cobb angle between the preoperative standing and flexibility test radiographs (such as supine lateral bending radiographs [7–9], fulcrum bending radiographs [6, 10], push-prone radiographs [8, 11], traction radiographs [5, 8, 10]) by the preoperative standing Cobb angle. Some studies have reported a strong correlation between flexibility and the postoperative correction rate [8, 12–14].

Existing techniques for assessing flexibility either depend on the patient’s voluntary effort or physician experience, but inherent variability exists in the subjective nature of these methods [15, 16]. To minimize the influence of the technique used or patient-related factors, supine radiographs have been proposed as an alternative for assessing curve flexibility. Cheung et al. found that the supine Cobb angle is significantly correlated with the immediate in-brace Cobb angle and that supine radiographs could be used to predict the immediate outcome of bracing a scoliotic spine [17]. In subsequent studies, supine flexibility was successfully used to predict curve progression in AIS patients undergoing underarm bracing and guide bracing treatment [18, 19]. In a study on operative cases of AIS, Cheh et al. showed that preoperative supine radiographs could accurately predict the Cobb angle on side-bending radiographs and concluded that preoperative supine radiographs could be used as an adjunct to predict the curve type, flexibility, and spinal structure with the advantage of being reproducible and relatively easy to assess [16]. Using a similar method, Ramchandran et al. reported a correlation between supine and supine lateral-bending radiographs when measuring the main thoracic (MT) and thoracolumbar (TL) curves and concluded that preoperative supine radiographs were highly predictive of side-bending radiographs for assessing curve flexibility in people suffering from AIS [15].

Although previous studies reported on the effectiveness of using supine radiographs for determining curve flexibility, to the authors’ knowledge, no publications have examined the use of preoperative supine flexibility for predicting the postoperative correction rate in AIS patients who underwent surgical correction. The purpose of this study was to determine the correlation between supine flexibility and postoperative correction in AIS patients. If a significant correlation was observed, a regression model would be used to examine whether the assessment of supine flexibility could predict the postoperative correction rate.

## Methods

This study was a retrospective analysis of patients with AIS who underwent surgical treatment between 2018 and 2020 at Peking Union Medical College Hospital. Ethical approval for the study protocols was obtained from the Institutional Review Board of Peking Union Medical College Hospital (No. I-22PJ124). The inclusion criteria were as follows: (1) diagnosed with AIS aged 11–18 years old; (2) displayed a single curve; (3) third-generation spinal instrumentation was used in the correction surgery; and (4) fusion levels were determined according to the PUMC operative classification system [20]. Patients with AIS were excluded from the study if (1) the patient had a history of spinal surgery; (2) the patient displayed double curvature of the spine; (3) the patient underwent spinal osteotomy before fusion; or (4) image data were incomplete or image quality did not meet the study requirements. According to the location of the apex vertebrae, the subjects were divided into two groups: the thoracic curve group (T11 and above) and the lumbar curve group (T12 and below) [19].

**Fig. 1:**
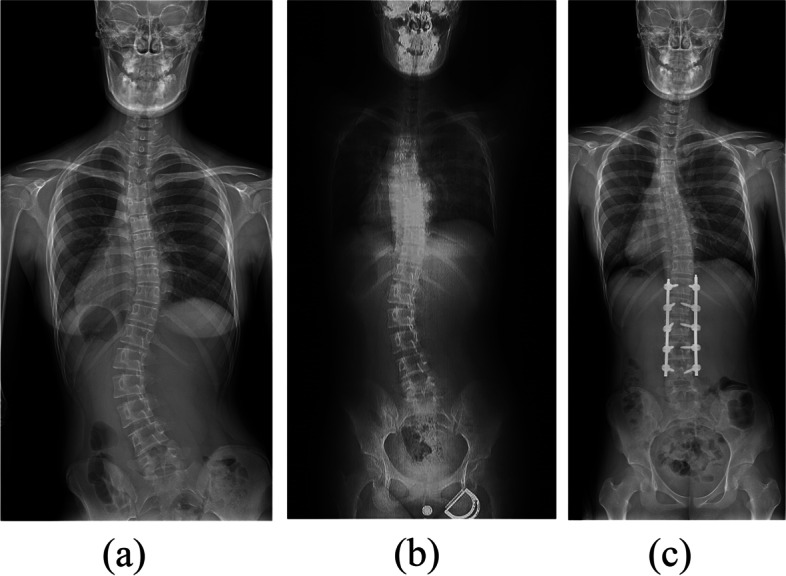
A 16-year-old girl with a (**a**) preoperative standing Cobb angle of 43.7°, (**b**) preoperative supine Cobb angle of 28.7° and supine flexibility of 34.3%, and (**c**) postoperative standing Cobb angle of 7.3° and correction rate of 83.3%

**Fig. 2:**
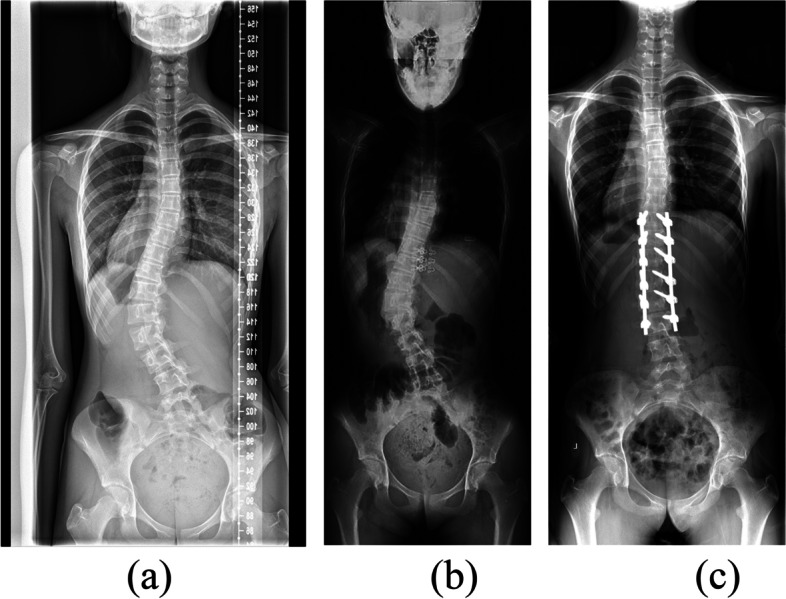
A 14-year-old girl with a (**a**) preoperative standing Cobb angle of 44.9°, (**b**) preoperative supine Cobb angle of 40.8° and supine flexibility of 9.1%, and (**c**) postoperative standing Cobb angle of 13.9° and correction rate of 69.0%

A total of 41 AIS patients were retrospectively enrolled in this study. The thoracic curve group consisted of 24 AIS patients (18 girls and 6 boys, with an average age of 14.6 ± 1.9 years), and the lumbar curve group consisted of 17 AIS patients (17 girls and 0 boys, with an average age of 14.5 ± 1.4 years). For all AIS patients, preoperative and postoperative standing radiographs and preoperative computed tomography (CT) images (size: 512 × 512, slice thickness < 5 mm) of the entire spine were collected. Because the archives of Peking Union Medical College Hospital did not have preoperative supine radiographs for the patients, CT images were transformed into supine X-ray-like images using the digitally reconstructed radiograph (DRR) technique [21, 22]. Specifically, CT scan images were projected onto the reference plane (coronal plane), and the window widths and levels were adjusted to improve image contrast using Python programming software (open source, version 3.9) [23]. The coronal Cobb angle was measured on preoperative standing radiographs, postoperative standing radiographs, and preoperative supine radiographs. All radiographs were measured by four independent observers using the image analysis software Digimizer 5.8.0 (MedCalc Software Ltd, Ostend, Belgium), and the mean of the four measurements was reported. Two typical cases are shown in Figs. 1 and 2. Based on the measured Cobb angle, the supine flexibility and postoperative correction rate were calculated by Eqs. (1) and (2):

1 $$Supine flexibility=\frac{(\mathrm{Preoperative standing Cobb angle }-\mathrm{ Preoperative Supine Cobb angle})}{\mathrm{ Preoperative standing Cobb angle}}* 100\mathrm{\%}$$

2 $$Postoperative correction rate=\frac{(\mathrm{Preoperative standing Cobb angle }-\mathrm{ Postoperative standing Cobb angle})}{\mathrm{Preoperative standing Cobb angle}}* 100\mathrm{\%}$$

SPSS 13.0 (SPSS, Inc., Chicago, IL) was used for data analysis. The reliability and variability of Cobb angle measurements were assessed by interobserver variability analysis and were expressed as the mean absolute difference (MAD) and the standard deviation (SD). T tests were used to assess differences in supine flexibility and postoperative correction between the groups, with *p* < 0.05 indicating a significant difference. Pearson’s product-moment correlation analysis was used to determine the correlation between supine flexibility and postoperative correction. The strength of the correlation was considered weak if the correlation coefficient (*r* value) was less than 0.39, moderate if the coefficient was between 0.40 and 0.59, strong if the coefficient was between 0.60 and 0.79, and very strong if the coefficient was between 0.80 and 1.00 [17, 24]. A linear regression model was used to determine whether the assessment of supine flexibility could predict the postoperative correction rate.

## Results

Table 1 shows the Cobb angles for the different curve types. For the thoracic curve group, the mean preoperative standing Cobb angle was 45.4 ± 14.0°, the mean preoperative supine Cobb angle was 35.4 ± 11.5° and the postoperative standing Cobb angle was 14.2 ± 5.5°. For the lumbar curve group, the mean preoperative standing Cobb angle was 42.2 ± 10.2°, the mean preoperative supine Cobb angle was 31.8 ± 9.2°, and the postoperative standing Cobb angle was 10.0 ± 3.9°. For all groups, the postoperative standing Cobb angle was significantly smaller than the preoperative Cobb angle (standing and supine).

**Table 1:** Preoperative standing Cobb angle, preoperative supine Cobb angle and postoperative standing Cobb angle

Group	Preoperative standing Cobb angle Mean ± SD (°)	Preoperative supine Cobb angle Mean ± SD (°)	Postoperative standing Cobb angle Mean ± SD (°)
Thoracic curve (*N* = 24)	45.4 ± 14.0^*^	35.4 ± 11.5^*^	14.2 ± 5.5
Lumbar curve (*N* = 17)	42.2 ± 10.2^*^	31.8 ± 9.2^*^	10.0 ± 3.9

*SD* Standard deviation, *N* Number of patients

^*^significant difference with the postoperative standing Cobb angle (*p* < 0.05)

Table 2 shows that the average MAD was 3.0° (2.1°-4.5°) for the preoperative standing Cobb angle, 5.3° (4.0°-6.9°) for the preoperative supine Cobb angle and 2.4° (1.8°-3.1°) for the postoperative standing Cobb angle. The SDs for the above three measurements were 4.9°, 6.0° and 3.1°, respectively.

**Table 2:** Interobserver variability analysis of Cobb angle measurements

	MAD (Observer 1)	MAD (Observer 2)	MAD (Observer 3)		MAD (Observer 4)		SD
Preoperative standing Cobb angle (°)	2.4	4.5		2.1		2.9	4.9
Preoperative supine Cobb angle (°)	4.8	6.9		5.3		4.0	6.0
Postoperative standing Cobb angle(°)	1.8	3.1		2.6		2.0	3.1

*SD* Standard deviation, *MAD* Mean absolute difference

A comparison between spinal supine flexibility and postoperative correction is shown in Table 3. For the thoracic curve group, the mean supine flexibility was 21.4 ± 13.1%, and the mean correction rate was 68.4 ± 9.7%; for the lumbar curve group, the values were 24.6 ± 11.3% and 76.1 ± 6.9%, respectively. For all groups, the supine flexibility was significantly lower than the correction rate (*p* < 0.05).

**Table 3:** Spinal supine flexibility and postoperative correction

Group	Supine flexibility Mean ± SD (%)	Correction rate Mean ± SD (%)
Thoracic curve (*N* = 24)	21.4 ± 13.1^*^	68.4 ± 9.7
Lumbar curve (*N* = 17)	24.6 ± 11.3^*^	76.1 ± 6.9

*SD* Standard deviation, *N* Number of patients

^*^significant difference with the correction rate (*p* < 0.05)

**Fig. 3:**
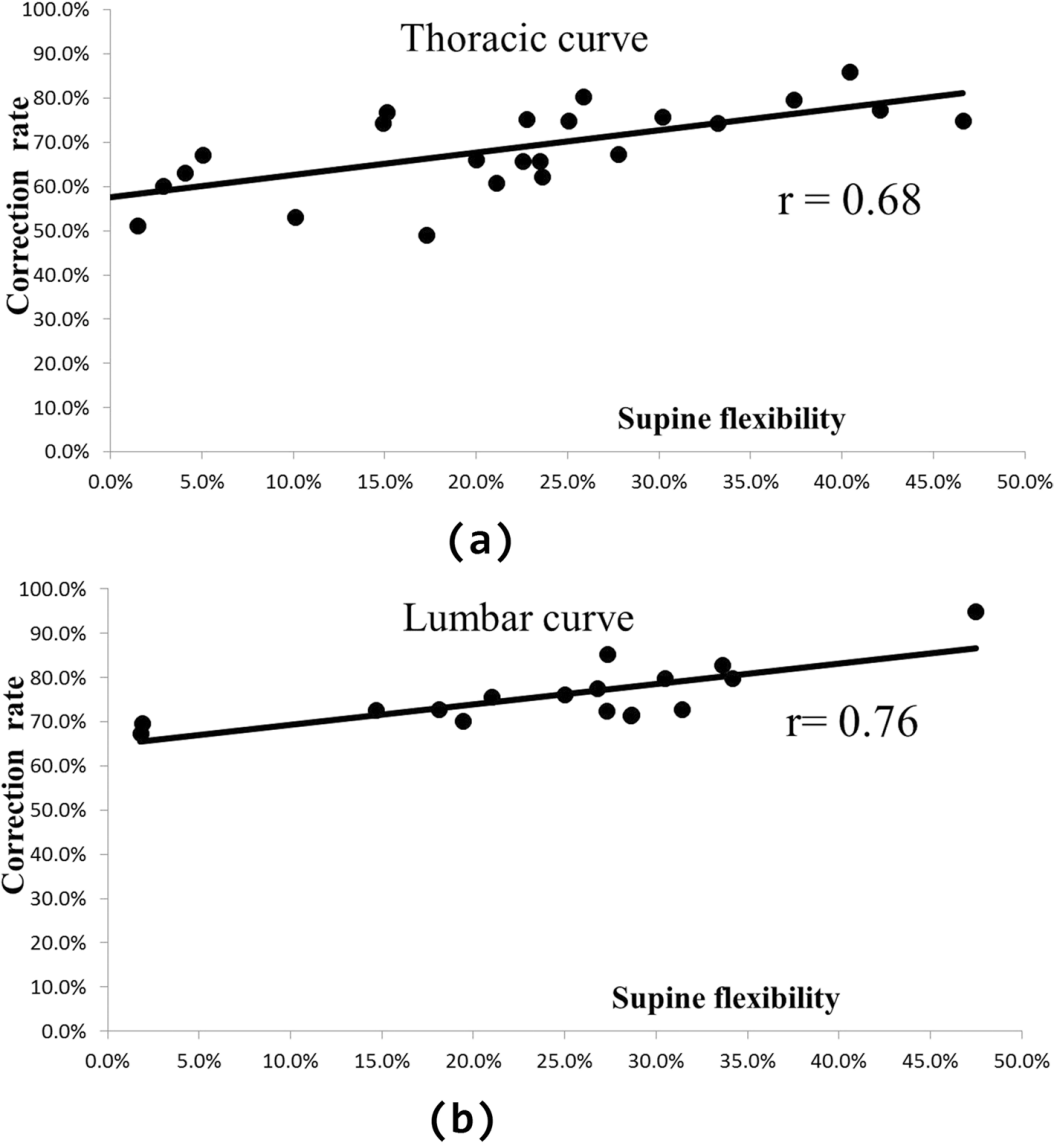
Correlation between supine flexibility and postoperative correction: (**a**) thoracic curve group, (**b**) lumbar curve group

Supine flexibility was strongly correlated with the postoperative correction rate, with an *r* value of 0.68 for the thoracic curve group (Fig. 3a) and 0.76 for the lumbar curve group (Fig. 3b). The univariate regression model was calculated by Eqs. (3) and (4):

For the thoracic curve group:

3 $$\mathrm{Correction rate }= (0.5044 *\mathrm{ Supine flexibility }+ 0.5767) * 100\mathrm{\%}$$

For the lumbar curve group:

4 $$\mathrm{Correction rate }= (0.4622 *\mathrm{ Supine flexibility }+ 0.6473) * 100\mathrm{\%}$$

The model predicted variances of 46.7% (thoracic curve group) and 57.4% (lumbar curve group) indicated a good fit for the data (F = 19.49 for thoracic curve group and F = 19.43 for lumbar curve group, *P* < 0.001).

## Discussions

To minimize the effect of radiographic technique or patient-related factors, supine radiographs have been proposed as an alternative method for assessing curve flexibility. In our study, a supine X-ray-like image was generated by applying the method of projecting CT scan data onto one reference plane (coronal plane). The above technology was called the digitally reconstructed radiograph technique, which is a mature technique that has been used successfully in spine measurement [21–23, 25]. Prost et al. showed that Cobb angles measured in supine images reconstructed from CT images of the spine could render similar information on flexibility and type of curves as bending radiographs [26]. Because spinal flexibility is often used to predict surgical correction rates, we conducted this study to analyse whether supine flexibility could predict the extent of postoperative correction in AIS patients.

Cheung et al. reported a mean difference of 3.6° between the supine and immediate in-brace Cobb angles, which indicated that the supine Cobb angle can be used to predict in-brace correction [17]. In a study on operative cases of AIS, Fei et al. showed that the mean differences between the fulcrum bending, supine lateral bending, suspension traction and postoperative Cobb angles were 2.3°, 7.4° and 13.4°, respectively [14]. However, Cheung et al. reported that there was a significant difference between the fulcrum bending Cobb (28°) and postoperative Cobb angles at 1 month (15°) and 2 years after surgery (16°) [13]. Elysee et al. investigated the correlation between preoperative supine imaging and postoperative alignment in adult spinal deformity (ASD) rather than AIS and reported that the difference between the supine and preoperative Cobb angles was 15.1° [27]. In our study, the mean difference between the supine and postoperative Cobb angles was 21.2° for the thoracic curve and 21.8° for the lumbar curve. Due to the lack of some active or passive bending forces, the above differences were larger than those in previous studies. As significant differences were found between the supine and postoperative Cobb angles (*p* < 0.05), the use of a supine Cobb angle alone cannot predict the extent of surgical correction.

Previous studies reported that the Cobb angle decreased on the coronal plane when the subject was transferred from a standing to supine posture [28, 29]. The difference between Cobb angles in the standing and supine positions was evaluated to analyse supine flexibility. Fei et al. reported no significant difference between fulcrum bending flexibility (75%) and surgical correction rate (80%), while supine lateral bending flexibility (63%) and suspension traction flexibility (50%) were significantly lower than the correction rate [14]. Cheung et al. reported that the fulcrum flexibility rate was 51% compared to correction rates of 72% and 70% at 1 month and 2 years after surgery [13]. In our study, the supine flexibility was significantly lower than the postoperative correction rate (*p* < 0.05), and the difference (47% for thoracic curve and 51.5% for lumbar curve) between them was larger than that in previous studies. However, there was a strong correlation between supine flexibility and the correction rate for the thoracic curve group and the lumbar curve group. The relationship between supine flexibility and postoperative correction rate can be expressed by a linear regression model. The lower correlation for the thoracic curve group may be explained by the more complex surgery, such as requiring interference with the rib cage.

Supine side bending radiographs are considered the standard method for determining curve flexibility [8–10, 15]. Cheh et al. and Ramchandran et al. showed that a single preoperative supine radiograph was highly predictive of side-bending radiographs and could be used as an adjunct to predict curve flexibility [15, 16]. Prost et al. compared Cobb angles of the main and minor curves in bending radiographs and supine images reconstructed from CT images and observed a significant correlation between bending radiographs and supine images [26]. Our results showed that supine flexibility can be used to predict the extent of postoperative correction in AIS patients. Accordingly, supine radiographs may be another useful technique for assessing flexibility and predicting postoperative correction since there is minimal impact from differences in radiographic techniques or patient-related factors. In the clinic, if a CT scan or a supine radiograph had been performed, bending radiographs might no longer be necessary.

This study has some limitations. (1) Supine flexibility was used in this study to predict postoperative correction in patients suffering from AIS. Due to the small sample size, an external validation study based on a separate large database may be needed to improve the accuracy of the prediction model. In addition, the findings may need to be further validated in patients with characteristics other than those included in this study. (2) Given the small population in this study, patients were not grouped or analysed according to sex, age or magnitude of the scoliotic curve. (3) Because the archives of Peking Union Medical College Hospital did not have the patients’ preoperative supine radiographs, supine radiographs were generated by projecting CT images. The prediction model presented in this study may need to be further verified using supine X-ray radiographs. (4) Although a strong correlation was shown between supine flexibility and postoperative correction, objective guidelines are not available for surgeons to utilize supine flexibility when planning the surgical approach. This may be considered in future research.

## Conclusions

This study found that supine flexibility can predict postoperative correction in AIS patients. In clinical practice, it may be possible to use supine radiographs in place of some existing flexibility test techniques.

## Data Availability

The data and materials supporting the conclusions of this article are included within the article. The raw data underlying the conclusions made in this study can be inquired to the corresponding author.

## Abbreviations

AIS: Adolescent idiopathic scoliosis
ASD: Adult spinal deformity
CT: Computed tomography
DRR: Digitally reconstructed radiograph
MAD: Mean absolute difference
MT: Main thoracic
N: Number of patients
SD: Standard deviation
TL: Thoracolumbar
